# Allyl Isothiocyanate Ameliorates Dextran Sodium Sulfate-Induced Colitis in Mouse by Enhancing Tight Junction and Mucin Expression

**DOI:** 10.3390/ijms19072025

**Published:** 2018-07-12

**Authors:** Min Woo Kim, Seungho Choi, Sun Yeou Kim, Yeo Sung Yoon, Ju-Hee Kang, Seung Hyun Oh

**Affiliations:** 1 Department of Anatomy and Cell Biology, College of Veterinary Medicine, Seoul National University, 08826 Seoul, Korea; eastsea1203@gmail.com (M.W.K.); schoi790227@gmail.com (S.C.); ysyoon@snu.ac.kr (Y.S.Y.); 2 Gachon Institute of Pharmaceutical Sciences, Gachon University, 21936 Incheon, Korea; sunnykim@gachon.ac.kr

**Keywords:** allyl isothiocyanate (AITC), *Wasabia japonica*, intestinal epithelial barrier, tight junction, mucin, mucin 2 (MUC2), goblet cell, dextran sodium sulfate (DSS), colitis

## Abstract

Inflammatory bowel disease (IBD) is characterized by chronic or recurrent inflammation of the gastrointestinal tract. Even though the current strategies to treat IBD include anti-inflammatory drugs and immune modulators, these treatments have side-effects. New strategies are, therefore, required to overcome the limitations of the therapies. In this study, we investigated the anti-colitic effects of allyl isothiocyanate (AITC), which is an active ingredient present in *Wasabia japonica*. The DSS-induced colitis model in the mouse was used to mimic human IBD and we observed that AITC treatment ameliorated the severity of colitis. We further studied the mechanism involved to ameliorate the colitis. To investigate the involvement of AITC on the intestinal barrier function, the effect on the intercellular tight junction was evaluated in the Caco-2 cell line while mucin expression was assessed in the LS174T cell line. AITC positively regulated tight junction proteins and mucin 2 (MUC2) against DSS-induced damage or depletion. Our data of in vivo studies were also consistent with the in vitro results. Furthermore, we observed that MUC2 increased by AITC is dependent on ERK signaling. In conclusion, we propose that AITC can be considered as a new strategy for treating IBD by modulating tight junction proteins and mucin.

## 1. Introduction

Inflammatory bowel diseases (IBD) including Crohn’s disease (CD) and ulcerative colitis (UC) are characterized by chronic or recurrent inflammation of the gastrointestinal tract [1]. Although the etiology of IBD is poorly understood, a previous study reported that its pathogenesis is related to the dysfunction of the intestinal epithelial barrier [2]. In healthy people, the intestinal epithelial barrier (including the intercellular tight junction and mucus layer) is selectively permeable to the intestinal contents, which prevents various antigens and microorganism from entering the host. In a pathological status of IBD, a dysfunctional intestinal epithelial barrier leads to increased intestinal permeability, which permits the passage of luminal antigens and bacteria and induces a host immune response [3]. A previous human study has reported that IBD patients have a dysfunctional intestinal epithelial barrier with increased intestinal permeability [4].

The dextran sodium sulfate (DSS)-induced colitis model in the mouse, which is one of the most widely used models for studying IBD, has revealed various aspects of IBD including the pathogenesis, immunology, genetic background, role of microbiome in IBD, and bowel malignancy secondary to IBD [5]. Even though the DSS-induced colitis model is unable to reflect all characters of human IBD, it is widely used because of similar histological features, rapid onset, simplicity, reproducibility, and controllability [6]. In addition, DSS disturbs the intestinal epithelial barrier function by decreasing tight junction proteins and mucin depletion [5,7,8].

The intestinal tight junction protein complexes connects the atypical membranes between intestinal epithelial cells and plays a role as a physical barrier, which provides selective permeability [9]. Defects in the tight junction results in increased gut permeability, which leads to an increased entrance of luminal antigens and contributes to intestinal inflammation [10]. The mucus layer is another physical barrier that protects the intestinal epithelium from harmful agents and bacteria. Mucin, which is the main component of the mucus layer, is produced by the goblet cells and mucin 2 (MUC2) is a major isotype in both human and mouse colon tissue [11,12]. *Muc2* knockout mice are more susceptible to DSS-induced colitis. Spontaneous colitis has also been observed without colitis induction [13], which emphasizes the importance of MUC2 for an intestinal barrier function. MUC2 depletion has also been reported as a characteristic of UC patients [14], which indicates that mucin depletion is closely related to human IBD. Irrespective of whether intestinal barrier dysfunction is the primary cause of inflammation or is a consequence secondary to inflammatory cytokines in human IBD, dysfunctional intestinal barrier augments the pathophysiological state of IBD [15]. Therefore, modulating intercellular tight junctions and mucin expression can be effective targets of novel therapeutic strategies for IBD.

The cruciferaceae family vegetable *Wasabia japonica* (WJ) is a popular traditional pungent spice in Asia (including Korea and Japan) and is used to treat rheumatic arthralgia by enhancing the blood circulation and pain relief [16]. It has been reported that a peculiar pungent taste of WJ and other cruciferous plants is derived from the breakdown products of glucosinolates [17]. Glucosinolates have a central thiocyanate group attached to d-glucose and myrosinase converts glucosinolates to glucose and an aglycone, which, in turn, is converted to an isothiocyanate [18]. Various isothiocyanates have been identified in WJ, which is the most abundant allyl isothiocyanate (AITC) [19]. AITC has been reported for its various biological effects including anti-cancer [20], anti-inflammatory [21,22], anti-angiogenic [23], anti-fungal [24], and anti-bacterial effects [25].

In our previous study, we determined that WJ is a functional food that prevents colitis without identifying a specific ingredient that mediates the anti-colitic effect [26]. Davaatseren et al. reported that AITC ameliorates DSS-induced colitis by attenuating angiogenesis and inflammation in mice [27]. Nevertheless, the extent of angiogenesis and inflammation can be affected not only by anti-angiogenic and anti-inflammatory effects of AITC, but also by the severity of the initial intestinal epithelial damage by DSS treatment. To our knowledge, the effects of AITC on the intestinal epithelial have never been explored before. We, therefore, undertook to investigate whether AITC protects the intestinal epithelium from DSS-induced colitis in mice by the modulation of the intestinal barrier including the tight junction and mucin expression.

## 2. Results

### 2.1. AITC Ameliorates DSS-Induced Colitis in Mice

**Figure 1:**
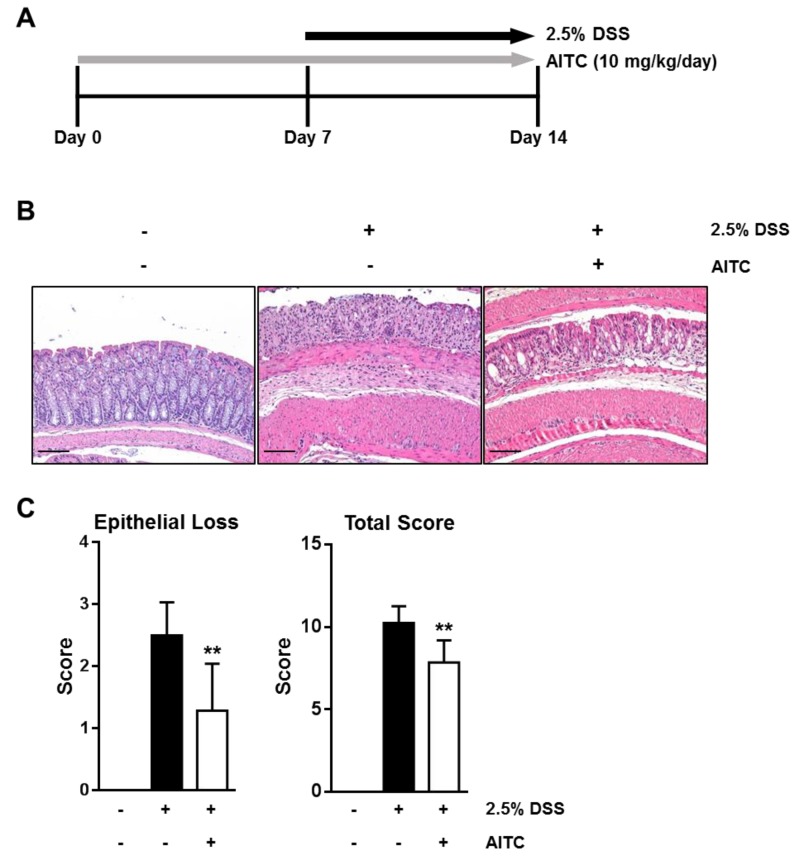
Protective effects of AITC against DSS-induced colitis in the mouse. (**A**) Animal experiment design for AITC treatment and colitis induction; (**B**) Histological figure of colon stained with H & E (Scale bar 100 μm); and (**C**) To evaluate severity of DSS-induced colitis, a histological scoring system was used. Numerical data were shown as a mean ± standard deviation (*t*-test was used for statistical analysis, ** *p* < 0.01).

To investigate whether AITC ameliorates DSS-induced colitis in vivo, AITC (10 mg/kg/day) was administered orally for seven days before DSS treatment. Treated and untreated mice were then exposed to 2.5% DSS in drinking water for seven days (Figure 1A). After sacrifice, the colon tissues were fixed and stained with hematoxylin and eosin (H & E) and microscopically examined. As shown in Figure 1B, 2.5% DSS treatment effectively induced colitis, which is characterized by widely distributed intestinal epithelial damage, goblet cell depletion, inflammatory cell infiltration, and edema. In contrast, the AITC treated group showed a comparatively intact intestinal epithelium with a larger number of goblet cells (Figure 1B). To evaluate the severity of DSS-induced colitis, we analyzed the colon tissues using a histological scoring system with respect to epithelial cell loss, crypt damage, and inflammation [28]. Our results show that the AITC treated group has a significantly lower score compared with the DSS-only treated group (Figure 1C, right). These results indicate that AITC treatment ameliorates the severity of DSS-induced colitis. Furthermore, we observed a significant difference in the surface epithelial cell loss between the AITC treated group and the DSS-only treated group (Figure 1C, left), which indicates that AITC may protect the intestinal epithelium through direct effects on the epithelium regardless of its anti-inflammatory effects and/or anti-angiogenic effects. We, therefore, hypothesized that AITC has a protective effect on DSS-induced colitis by regulating the intestinal epithelial barrier.

### 2.2. AITC Mildly Attenuates the LPS-Induced Inflammatory Signaling Pathway in RAW 264.7 Cells

**Figure 2:**
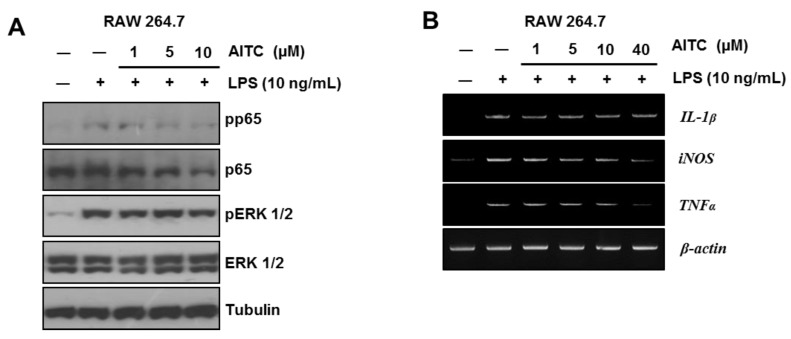
AITC mildly ameliorates the LPS-induced inflammatory signaling pathway in the RAW 264.7 murine macrophage cell line. (**A**) Western blot analysis was performed to determine the LPS-induced phosphorylation level of p65 and ERK ½; and (**B**) RT-PCR analysis was performed to evaluate the mRNA expression level of inflammatory cytokines (IL-1β and TNF-α) and iNOS.

Previous studies have reported the anti-inflammatory effects of AITC [21,22]. We confirmed the anti-inflammatory effects of AITC using the RAW 264.7 cell line that originates from mouse ascites. To induce inflammation and evaluate the effects of AITC, cells were exposed to lipopolysaccharides (LPS) (10 ng/mL) for six hours after pretreatment of AITC (1 μM, 5 μM, or 10 μM) for 18 h. We confirmed the main pathways of TLR4-mediated inflammatory signaling, which include the extracellular signal-related kinase (ERK) and the nuclear factor kappa-light-chain-enhancer of activated B cells (NF-κB). Consistent with previous reports [21,22], AITC mildly attenuated the phosphorylation levels of p65 and ERK 1/2 at 10 μM (Figure 2A). Besides ERK and NF-κB signaling, the LPS-induced mRNA expressions of interleukin-1β (IL-1β), inducible nitric oxide synthase (iNOS), and tumor necrosis factor-α (TNF-α) were also mildly decreased by AITC treatment (Figure 2B). Therefore, we believe that the anti-colitic effects of AITC may not be fully dependent on the anti-inflammatory effects of AITC.

### 2.3. The Effects of AITC on the Intestianl Intercellular Tight Junction

**Figure 3:**
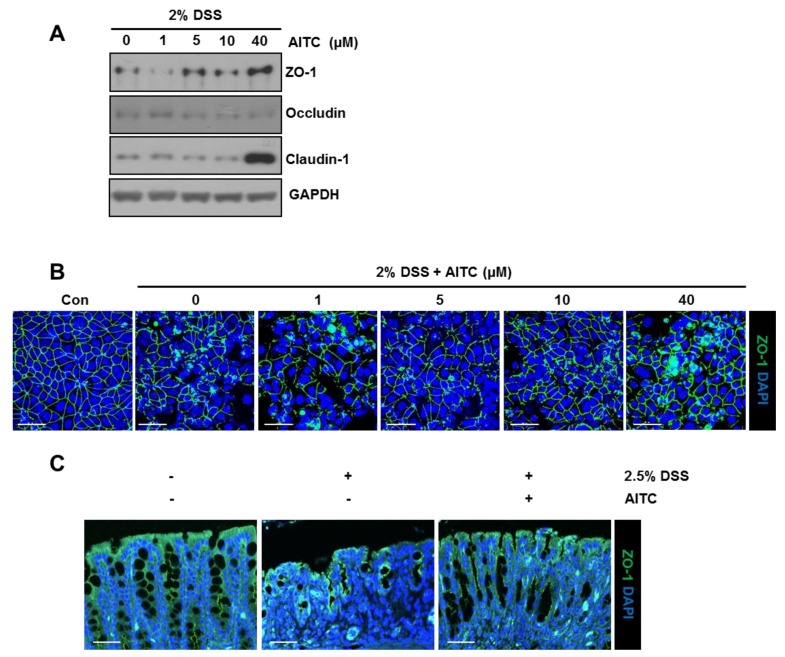
AITC increases tight junction proteins and inhibits a DSS-induced tight junction disruption in the Caco-2 cell line and mouse. (**A**) Effects of AITC treatment (1, 5, 10, or 40 μM) with 2% DSS on the expression of tight junction proteins analyzed in Caco-2 by Western blotting; (**B**) Protective effects of AITC pretreatment (1, 5, 10, or 40 μM) against the DSS-induced damage examined by immuno-fluorescent ZO-1 staining (ZO-1: **green**, DAPI: **blue**, Scale bar 50 μm); and (**C**) Colon tissues from mice were examined by ZO-1 staining (Scale bar 50 μm).

The human colon carcinoma cell line (Caco-2) is derived from intestinal epithelial cells and forms a monolayer containing intercellular tight junctions when cultured to 100% confluence. Therefore, this cell line is broadly used as an intestinal barrier model [29]. To explore the effect of AITC on the expression of tight junction proteins, Caco-2 monolayers were exposed to AITC (1, 5, 10, or 40 μM) in the presence of 2% DSS. We observed that exposure to AITC increases the tight junction protein levels such as zonula occludens-1 (ZO-1) and claudin-1 (except occludin) when compared to the control (Figure 3A). Simultaneously, we also treated 2% DSS to a Caco-2 monolayer for 48 h with or without AITC (1, 5, 10, or 40 μM) pretreatment to study the protective effects of AITC on a tight junction. After all the relevant treatments, the Caco-2 monolayers were stained using a ZO-1 specific antibody to visualize an intercellular tight junction (green color). As shown in Figure 3B, 2% DSS treatment resulted in tight junction disruption, but AITC pretreatment at doses of 5 μM, 10 μM, and 40 μM protected the tight junction structure from a DSS-induced tight disruption. Furthermore, colon tissue from the in vivo study were stained with a ZO-1 specific antibody to verify the protective effects on the tight junction. The colons of the DSS-only treated group showed discontinuous and damaged tight junctions as compared to the control group. Conversely, colons of the AITC group showed more intact and continuous tight junction structures as compared to the DSS-only treated group (Figure 3C). These results explain that exposure to AITC increases the expression of tight junction proteins under stress conditions induced by DSS and protects the tight junction structure from DSS-induced damage in vitro and in vivo.

### 2.4. The Effects of AITC on Mucin Expression in Goblet Cells

**Figure 4:**
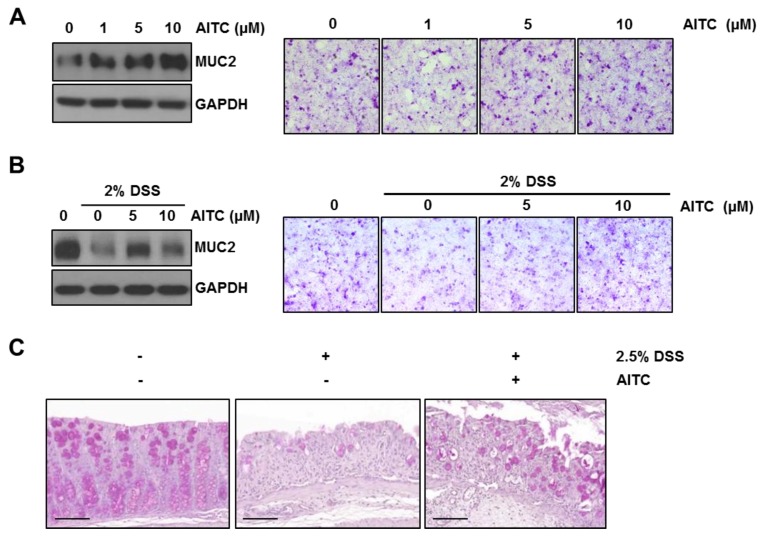
AITC positively regulates MUC2 expression and attenuates DSS-induced mucin depletion in the LS174T cell line and in mice. To evaluate mucin expression, Western blot analysis using the MUC2 specific antibody and PAS staining were performed (PAS staining, mucin: magenta color). (**A**) AITC treatment (1, 5, or 10 μM) for 12 h increased MUC2 in LS174T (Right, 200× magnification); (**B**) AITC pretreatment ameliorated MUC2 decrease by DSS in LS174T (Right, 200× magnification); and (**C**) Mucin and goblet cell depletion in DSS-induced colitis was protected by AITC treatment. Colon tissues were stained with PAS staining (Scale bar 100 μm).

The intestinal epithelial goblet cells release mucin into the intestinal lumen, which forms a mucus layer to protect the intestinal epithelium from luminal contents. Since the LS174T cell line is an intestinal epithelial cell line showing goblet cell-like characteristics and is used to mimic goblet cells [30], this cell line was, therefore, used to study whether AITC regulates the mucin expression and attenuates DSS-induced mucin decrease. To investigate the effect of AITC on MUC2 expression, cells were first treated with AITC (1, 5, or 10 μM) for 12 h. The MUC2 expression level was evaluated by Western blotting using an MUC2 specific antibody. Periodic acid-Schiff (PAS) staining (without counter staining) was used for the visualization of mucin in which the mucin produces a magenta color. We observed that AITC treatment enhanced the MUC2 expression in a dose-dependent manner (Figure 4A). Next, cells were pretreated with AITC (5 or 10 μM) for six hours, which was followed by 2% DSS treatment for 12 h. As shown in Figure 4B, 2% DSS effectively decreased MUC2 when compared with the control while AITC pretreatment resulted in the attenuation of DSS-induced MUC2 depletion at doses of 5 and 10 μM. To investigate the enhancement of mucin expression against DSS-induced colitis after exposure to AITC, the colon tissues were stained with PAS staining (Figure 4C). The DSS-only treated group showed a disruption of the mucus layer and very few goblet cells when compared with the control group. Goblet cells were markedly increased in numbers with strongly stained mucin granules within the cells in the AITC treated group. These results indicate that AITC positively regulates the mucin expression and, thereby, reverses DSS-induced mucin depletion.

### 2.5. AITC Enhances MUC2 Expression through the ERK-Dependent Pathway

**Figure 5:**
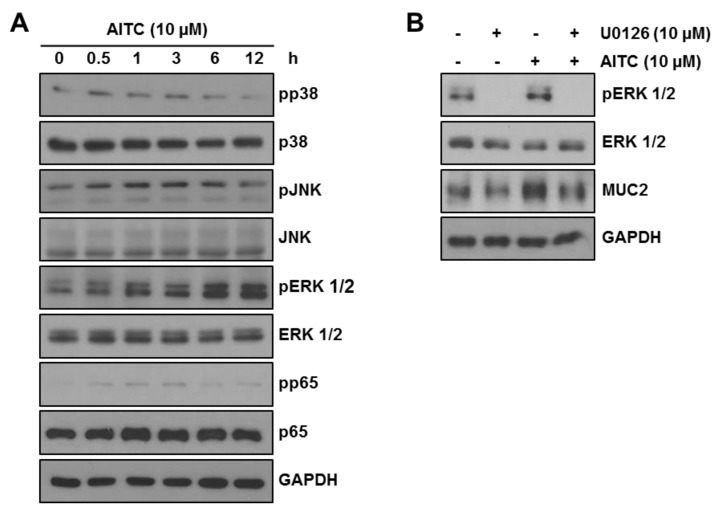
AITC increases MUC2 expression through the ERK-dependent pathway. (**A**) Phosphorylation of p38, JNK, ERK, and p65 was investigated after AITC 10 μM treatment (0.5, 1, 3, 6, or 12 h) by Western blotting; and (**B**) ERK activation and MUC2 expression were examined after treating AITC 10 μM and/or U0126 10 μM by Western blotting.

To determine the mode of action through which AITC increases MUC2 expression, we evaluated the effect on mitogen-activated protein kinase (MAPK) signaling pathways including p38, ERK, c-Jun N-terminal kinase (JNK), and NF-κB, which are the known MUC2 regulation pathways [31,32,33]. LS174T cells were treated with 10 μM AITC for the indicated time and phosphorylation of p38, ERK, JNK, and p65 was assessed by Western blotting. Phosphorylation of ERK increased in a time-dependent manner while those of p38, JNK, and p65 showed mild increases or no changes (Figure 5A). Therefore, we supposed that AITC increases the MUC2 expression through the ERK-dependent pathway in LS174T cells. To confirm the increase of MUC2 expression through the EKR pathway after AITC exposure, we used an ERK inhibitor (U0126) for pharmacological inhibition of ERK. As shown in Figure 5B, 10 μM U0126 effectively blocked ERK phosphorylation. We observed that treatment with U0126 alone showed no decrease in the MUC2 expression when compared with the control. However, increased MUC2 expression after exposure to AITC was reversed in the presence of U0126. These results strongly suggest that AITC-induced MUC2 expression is regulated through the ERK-dependent pathway while the basal level of MUC2 expression is not fully dependent on the ERK pathway.

## 3. Discussion

Even though some strategies to treat IBD exist such as anti-inflammatory drugs and immuno-modulators, the non-specificity of these treatments result in accompanying adverse side effects, which limits their usage [34]. The need for complementary and alternative medicine (CAM), which provides options to treat IBD, are, therefore, increasing. One of the most widely used CAM by IBD patients is herbal medicine, but, in many cases, the lack of scientific evidence threatens the safety of these therapeutics [35]. Therefore, the safety and effects of each active compound derived from natural products needs to be verified with scientific evidence.

In a previous study, we observed that *Wasabia japonica* (WJ) attenuates DSS-induced colitis in mice by exerting its anti-inflammatory effects. Furthermore, WJ coated with 5% Eudragit increases absorption in the colon, which increases the protective effects of WJ [26]. However, the active ingredient mediating the anti-colitic effects of WJ was not identified. AITC, which is one of the bioactive molecules present in WJ, is the most abundant isothiocyanate [19] that exerts various biological effects such as anti-cancer [20], anti-inflammatory [21,22], anti-angiogenic [23], anti-fungal [24], and anti-bacterial effects [25]. Therefore, hypothesizing that AITC would have a major role in the anti-colitic effects of WJ, this study investigated the protective effects of AITC against DSS-induced colitis and the potential mechanism involved. Although Davaatseren et al. have previously reported about the protective effects of AITC in DSS-induced colitis in mice, their focus was mainly on the anti-angiogenetic and anti-inflammatory effects [27]. Considering the pathogenesis of DSS-induced colitis, AITC may affect not only the severity of angiogenesis and inflammation, but also the severity of the initial epithelial damage induced by DSS. In the early stages of DSS-induced colitis, DSS induces the epithelial barrier damage resulting in leakage of luminal antigens, which initiate the inflammatory response [5]. The extent of early inflammation may, therefore, depend on the severity of the initial intestinal epithelial damage and barrier dysfunction attributed to DSS. However, inflammation induced by the initial barrier dysfunction also influences the intestinal barrier through inflammatory cytokines [9]. Considering the reciprocal relationship between the intestinal barrier dysfunction and inflammation, the mechanism of anti-colitic effects by AITC are not limited to anti-inflammation and anti-angiogenesis. In our animal study, we observed a significantly lower histological score of epithelial cell loss in the AITC treatment group than in the DSS only treated group, which implies that AITC likely affected the intestinal epithelium directly. This prompted us to investigate the effects of AITC on the intestinal barrier including the intercellular tight junction and mucus layer. To the best of our knowledge, there is no prior report on the relationship between AITC and the intestinal epithelial barrier. This study is the first to suggest that AITC ameliorates DSS-induced colitis by enhancing tight junction and mucin expression.

Consistent with previous studies on the anti-inflammatory effects of AITC [21,22], we observed that 10 μM AITC mildly attenuates the LPS (1 μg/mL)-induced inflammatory signaling in RAW 264.7 cells. Accordingly, we postulated that AITC attenuates the DSS-induced colitis not only through anti-inflammatory effects but also through other pathways.

One feature of IBD pathogenesis is a disrupted tight junction structure, which is also observed in the DSS-induced colitis model [8]. As shown in our animal study, AITC exposure ameliorates the tight junction disruption by DSS. Damage of the tight junction is mediated by the direct effect of DSS [36] or pro-inflammatory cytokines secondary to inflammation [9]. In the in vitro study using Caco-2 cells exposed to 2% DSS, we observed an increase in the expression of tight junction proteins (such as ZO-1 and claudin) in the presence of AITC. In addition, DSS-induced tight junction disruption was reversed by the AITC treatment without any influence in the pro-inflammatory cytokine levels. These data suggest that AITC positively regulates the tight junction expression and protects the tight junction from DSS-induced damage.

Mucin depletion is another feature of human IBD patients especially in ulcerative colitis [14]. Since the DSS-induced colitis model also shows goblet cell and mucin depletion [5], we investigated whether AITC can regulate mucin expression. Since MUC2 is a major mucin in both humans and mice [11,12], we focused on MUC2 expression among various mucin isotypes. In the in vitro studies using LS174T cells, exposure to AITC increases the MUC2 expression while treatment with 2% DSS decreased the MUC2 expression. However, AITC pretreatment rescued the DSS-induced MUC2 depletion. The in vivo PAS staining study also showed results consistent with the above observation. These results indicate that AITC alone increases mucin expression and also attenuates the DSS-induced mucin depletion. To determine the mechanism through which AITC increases MUC2 expression, we screened the p38, JNK, ERK, and p65 pathways since these pathways were previously reported to enhance MUC2 expression [31,32,33]. We found that AITC-induced dramatic activation of ERK while phosphorylation of p38, JNK, and p65 was mild. To confirm whether AITC enhances MUC2 expression through EKR phosphorylation, we pharmacologically inhibited ERK with U0126 and observed that the AITC-induced MUC2 increase was reversed by blocking the ERK pathway while MUC2 expression at the basal level remains unaffected by ERK inhibition. We believe that the basal expression of MUC2 may not be completely dependent on the ERK pathway and other pathways such as p38, p65, and JNK play a role in the expression of MUC2 when ERK activation is blocked.

In conclusion, our data indicates that AITC attenuates the severity of DSS-induced colitis in mice by enhancing the intestinal barrier including both tight junction and mucin expression. We propose that AITC should be considered a new agent that ameliorates IBD by targeting the intestinal barrier function.

## 4. Materials and Methods

### 4.1. Animals, Cells, and Materials

Female six-week-old C57BL/6 mice were purchased from the Charles River Laboratories (Wilmington, MA, USA). RAW 264.7 murine macrophages, Caco-2 human colon carcinoma cell line, and the LS174T human epithelial cell line were obtained from the Korea Cell Line Bank (Seoul, Korea). Cells were cultured in DMEM medium (Caco-2 and RAW 264.7) or RPMI 1640 medium (LS174T) supplemented with 10% fetal bovine serum (FBS) and penicillin/streptomycin (Welgene, Inc., Daegu, Korea). Dextran sodium sulfate (DSS) was purchased from MP Biomedicals (Santa Ana, CA, USA). LPS purified from *Escherichia coli* O111:B4 was purchased from Sigma-Aldrich (St. Louis, MO, USA). AITC from WJ was kindly provided by Sun Yeo Kim. U0126 was purchased from Calbiochem (San Diego, CA, USA). The ZO-1, occludin, and claudin-1 antibodies were purchased from Invitrogen (Carlsbad, CA, USA), and p65. GAPDH and MUC2 antibodies were purchased from Santa Cruz Biotechnology (Santa Cruz, CA, USA). Antibodies for phospho-p65 was purchased from the Cell Signaling Technology company (Danvers, MA, USA). Schiff’s reagent and periodic acid were purchased from Sigma-Aldrich (St. Louis, MO, USA).

### 4.2. DSS-Induced Mouse Colitis Model

All animal procedures were conducted according to a protocol approved by the Institutional Animal Care and Usage Committee (IACUC) [LCDI-2014-0054; 16 September 2014] at Gachon University in Incheon, Korea. The animal experiments were completed as previously described with several modifications [37]. Animals were randomly divided into three groups including (1) the vehicle-treated control (*n* = 5), (2) the 2.5% DSS only in drinking water for 7 days (*n* = 8), and (3) pretreatment with AITC 10 mg/kg/day p.o. for 7 days before exposure to 2.5% DSS in their drinking water for 7 days (*n* = 7). At the end of the experimental period, mice were euthanized by CO_2_ inhalation and colons were excised for histological evaluation.

### 4.3. Histological Analysis of Colitis

After excision, colons were Swiss-rolled and fixed in 10% neutral buffered formalin. After paraffin-embedding, colons were sectioned at 5 μM thickness and stained with hematoxylin and eosin (H & E). Histological scoring was done for the surface epithelial loss, crypt destruction, and inflammatory cell infiltration [28].

### 4.4. Western Blot Analysis

RAW 264.7 cells were pretreated with or without 1 μM to 10 μM of AITC for 18 h and then stimulated with LPS (10 ng/mL) for 6 h. Caco-2 cells were seeded and, after attaining 90% to 100% confluency, they were differentiated for 14 days. Afterward, they were incubated with 2% DSS in the absence or presence of AITC (1, 5, 10, and 40 μM) for 48 h. LS174T cells were treated with AITC (1, 5, or 10 μM) for 12 h. In the co-treatment schedule, LS174T cells were pretreated with or without AITC (5 or 10 μM) for 6 h, which was followed by 2% DSS treatment for 12 h. Preparation of whole-cell lysates, protein quantification, gel electrophoresis, and Western blotting was performed as described previously [37]. Protein concentrations were measured using a bicinchoninic acid protein assay (Pierce Biotechnology, Rockford, IL, USA). An equal amount of protein from cell lysates was resolved using sodium dodecyl sulfate-polyacrylamide gel electrophoresis and was immunoblotted with primary antibodies. Antibody binding to the membrane was detected using enhanced chemi-luminescence Western blotting detection reagents obtained from Absignal (Abclone, Seoul, Korea).

### 4.5. RNA Isolation and RT-PCR

To examine gene expression changes at the RNA level, a reverse transcriptase (RT)-polymerase chain reaction (PCR) was performed. First, total RNA was isolated using the TRIzol reagent (Invitrogen, Carlsbad, CA, USA). Complementary DNA (cDNA) was then synthesized from 2 μg of the RNA extracted from cells using the PrimeScript RT reagent Kit (TaKaRa, Shiga, Japan). cDNA was then amplified by PCR with mouse-specific primers: IL-1β, 5′-GCC TTG GGC CTC AAA GGA AAG AAT C-3′ (forward) and 5′-GGA AGA CAC AGA TTC CAT GGT GAA G-3′ (reverse), TNF-α, 5′-ATA GCT CCC AGA AAA GCA AGC-3′ (forward) and 5′-CAC CCC GAA GTT CAG TAG ACA-3′ (reverse), iNOS, 5′-GTG GTG ACA AGC ACA TTT GG-3′ (forward) and 5′-GGC TGG ACT TTT CAC TCT GC-3′ (reverse), and β-actin, 5′-TGG AAT CCT GTG GCA TCC ATG AAA C-3′ (forward) and 5′-TAA AAC GCA GCT CAG TAA CAG TCC G-3′ (reverse). After PCR, the resultant products were analyzed by separation on a 1.5% agarose gel in tris-acetate/ethylenediaminetetraacetic acid (EDTA) buffer.

### 4.6. Immunofluorescence Assay

To evaluate the effect of AITC on DSS-treated Caco-2, the cells were seeded onto coverslips placed in 12-well plates (Costar, Corning, NY, USA). Upon reaching 90 to 100% confluency, the Caco-2 cell monolayer was allowed to differentiate for an additional 14 days. Fully differentiated cell monolayers were incubated with or without 2.5% DSS for 48 h after pretreatment of AITC for 24 h. The cells were then fixed and permeabilized in a methanol: acetone (1:1) mixture at −20 °C for 20 min. Cells were incubated overnight with primary antibodies at 4 °C, which was followed by incubation with an FITC-labeled secondary antibody for 2 h at room temperature. Coverslips were then mounted with mounting medium containing 4,6-diamidino-2-phenylindole (DAPI) for nuclear counterstaining. Images were observed by confocal microscopy. FITC and DAPI images were taken from the same area. Colon tissues with 5 μm thickness were stained by the same method after deparaffinization and antigen retrieval.

### 4.7. Periodic Acid-Schiff (PAS) Staining

LS174T cells were seeded on coverslips. On the following day, each treatment was done as described above. Three washes of PBS (each wash for 5 min) was followed by methanol treatment for 10 min at −20 °C, which was followed by three washes of PBS. Cells were then incubated with periodic acid for 5 min at room temperature. After three washes of PBS, cells were treated to Schiff’s solution for 15 min at room temperature. Lastly, the coverslips were washed three times, dehydrated, and mounted on slide glasses for viewing.

## Abbreviations

IBD	inflammatory bowel disease
AITC	allyl isothiocyanate
MUC2	mucin 2
CD	Crohn’s disease
UC	ulcerative colitis
DSS	dextran sodium sulfate
H&E	hematoxylin and eosin
PAS	periodic acid-Schiff
LPS	lipopolysaccharide
ERK	extracellular signal-related kinase
NF-κB	nuclear factor kappa-light-chain-enhancer of activated B cells
IL-1β	interleukin-1β
TNF-α	tumor necrosis factor-α
iNOS	inducible nitric oxide synthase
ZO-1	zonula occludens-1
CAM	complementary and alternative medicine
WJ	*Wasabia japonica*
